# *In vitro* exposure to epididymal extracellular vesicles from normospermic domestic cats improves developmental potential of sperm from teratospermic cats

**DOI:** 10.3389/fvets.2025.1547175

**Published:** 2025-01-17

**Authors:** Danielle M. Sosnicki, Alexander J. Travis, Pierre Comizzoli

**Affiliations:** ^1^Department of Reproductive Sciences, Smithsonian’s National Zoo and Conservation Biology Institute, Washington, DC, United States; ^2^Baker Institute for Animal Health, Cornell University, Ithaca, NY, United States; ^3^Department of Public & Ecosystem Health, Cornell University, Ithaca, NY, United States

**Keywords:** extracellular vesicles, epididymis, sperm, teratospermia, developmental potential, domestic cat

## Abstract

We have previously reported a difference in the composition of epididymal extracellular vesicles (EVs) between normospermic and teratospermic domestic cats. The objective of the present study was to investigate whether the fertilizing ability or developmental potential of sperm from teratospermic cats could be improved after incubation with EVs isolated from normospermic cats. For each of 11 experimental replicates, pools of EVs were collected from the whole epididymides of 5 normospermic cats (normospermic EVs). Spermatozoa were also collected from the cauda epididymides of 2 teratospermic cats, pooled, and half was co-incubated with normospermic EVs for 1 h and 15 min prior to using the sperm for *in vitro* fertilization (IVF). The other half of the sperm was kept for 1 h and 15 min in the absence of EVs as a control group. We found no difference (*p* > 0.05) in sperm fertilizing ability, based on the percentage of cleaved embryos, after incubation with EVs (67.0%) and without EVs (60.6%). However, the developmental potential of teratospermic sperm, based on the proportion of embryos that reached the 8-cell stage or further, was better (*p* < 0.05) after co-incubation with EVs (58.4%) compared to the control group without EVs (47.2%). Additionally, the proportion of embryos that reached the blastocyst stage was better (*p* < 0.05) after co-incubation with EVs (30.7%) compared to the control group without EVs (19.9%). These findings can be used to improve the outcome of IVF with teratospermic males in domestic or wild felid species.

## 1 Introduction

Mammalian spermatozoa acquire the ability to be motile, fertilize an oocyte, and sustain successful embryo development during epididymal maturation, a process that occurs as sperm cells transit through the different milieus of the epididymis (1–3). During this process, sperm cells are transcriptionally and translationally silent (4, 5) so changes reflect post-translational modifications. In multiple animal species, extracellular vesicles (EVs) originating from the epididymal epithelium are known to transfer proteins, RNAs, lipids, and other key factors to sperm aiding in this full maturation (1, 6–11).

The domestic cat is an ideal model for functional studies in sperm, due to the very high prevalence of teratospermia (>60% abnormal sperm per ejaculate) found in this species (12, 13). This mimics the variation in sperm defects that also are observed in wild species within the Felidae family, such as cheetah, clouded leopard, and tiger (14–16). Many species of wild felids could benefit from improved sperm function during assisted reproductive technologies that are aimed at improving reproductive success needed for species conservation efforts.

In felids, even morphologically normal spermatozoa from teratospermic ejaculates lead to poor embryo development indicating that even cells that appear structurally normal suffer from functional defects that compromise their ability to lead to viable offspring (12, 13). We previously reported that there are at least 98 proteins found in epididymal EVs derived from normospermic cats that are not found in epididymal EVs from teratospermic cats (17). This led us to think that some of these proteins as well as other factors might be involved in fertilization and/or ensure the sperm capacity to sustain a successful embryo development (developmental potential). The underlying hypothesis was that some of the functional deficits in sperm from teratospermic cats are due to incomplete epididymal sperm maturation resulting from exposure to abnormal epididymal EVs. Therefore, the objective was to study if it was possible to restore some of the epididymal maturation process *in vitro* by exposing abnormal sperm to epididymal EVs from normospermic cats. Specifically, we examined the fertilizing ability and developmental potential of sperm from teratospermic cats after incubation with EVs from normospermic cats and *in vitro* fertilization (IVF).

## 2 Materials and methods

### 2.1 Animals and reagents

All reagents were from Sigma, unless otherwise noted. The study did not require the approval of the Animal Care and Use Committee of Smithsonian’s National Zoo and Conservation Biology Institute because cat testes and ovaries were collected at local veterinary clinics as byproducts from owner-requested routine orchiectomies and ovariohysterectomies. Tissues were stored and transported to the lab in phosphate buffered saline (PBS) at 4°C within 24 h of excision.

### 2.2 Oocyte collection and *in vitro* maturation

Six pairs of ovaries were used for each oocyte collection and subsequent IVF experiment (*n* = 11 experimental replicates; 479 total oocytes from 132 ovaries from 66 cats). Ovaries were minced in room temperature (~20–22°C) handling medium (Minimum Essential Medium (MEM) with Hank’s salt with glutamine (Gibco Laboratories), supplemented with 10 mM HEPES, 100 IU/mL penicillin, 100 μg/mL streptomycin, 1 mM pyruvate, and 0.1% bovine serum albumin), and Grade I immature oocytes were selected by identifying them as having homogeneous dark cytoplasm and several layers of intact cumulus cells. Selected oocytes were cultured at 38.5°C and 5% CO_2_ under mineral oil for 26–28 h in *in vitro* maturation (IVM) medium containing SAGE protein plus blastocyst medium (CooperSurgical Fertility Solutions) supplemented with 25 μg/mL porcine Follicle Stimulating Hormone (Sigma F2293) and 1 μg/mL ovine Luteinizing Hormone (National Hormone and Pituitary Program, United States), prior to *in vitro* fertilization.

### 2.3 Extracellular vesicle collection from normospermic cats

Epididymides were removed from testes and rinsed in PBS. A small number of spermatozoa were collected from the cauda and assessed via Coomassie staining to determine normospermic individuals (<60% abnormal spermatozoa). Whole epididymides (including caput, corpus, and cauda regions) from normospermic cats (*n* = 55 normospermic cats, with 5 males pooled in each of the 11 experimental replicates) were minced with a blade in cold Ham’s F-10 Nutrient Mix with HEPES (Gibco Laboratories) on the benchtop and luminal fluid was allowed to seep out for 10 min. Liquid was triturated and collected into 1.5 mL tubes. Serial centrifugations were performed at 700 *x g* for 10 min and 3,000 *x g* for 10 min to remove tissue debris and sperm cells, respectively. Samples were stored at −80°C until ultracentrifugation. Once EV suspensions were collected from 5 normospermic cats, samples were pooled together and ultracentrifuged at 100,000 *× g* for 2 h at 4°C to obtain a pellet of EVs derived from the whole epididymis. The pellet was resuspended in 50 μL of fresh Ham’s F-10 Nutrient Mix with HEPES and stored at −80°C. A Qubit Protein Assay Kit (Molecular Probes by Life Technologies) was used to assess the concentration of EVs in each pooled sample. Successful isolation of EVs using this method was previously confirmed via observations performed using a transmission electron microscope (Zeiss 10 CA Transmission Electron Microscope) at the University of Maryland Laboratory for Biological Ultrastructure, United States (8) with sizes of EVs ranging from 50 to 200 nm. Previous proteomic analyses of the isolated EVs revealed several known proteins associated with exosomes according to the International Society for Extracellular Vesicles (17, 18). Additional relevant data of collection can also be accessed in the EV-TRACK knowledgebase (EV-TRACK ID: EV200074) (19).^1^ For this study we use the operative term “extracellular vesicle” (EV) in accordance with the guidelines outlined by the Minimal Information for Studies of Extracellular Vesicles (MISEV2023) which suggests the use of this term when defining an isolated sample that has not been further analyzed for EV subtype (e.g., exosome, microvesicle, apoptotic vesicle, etc.) (18, 20).

### 2.4 Sperm collection from teratospermic cats and swim up procedure

**Figure 1:**
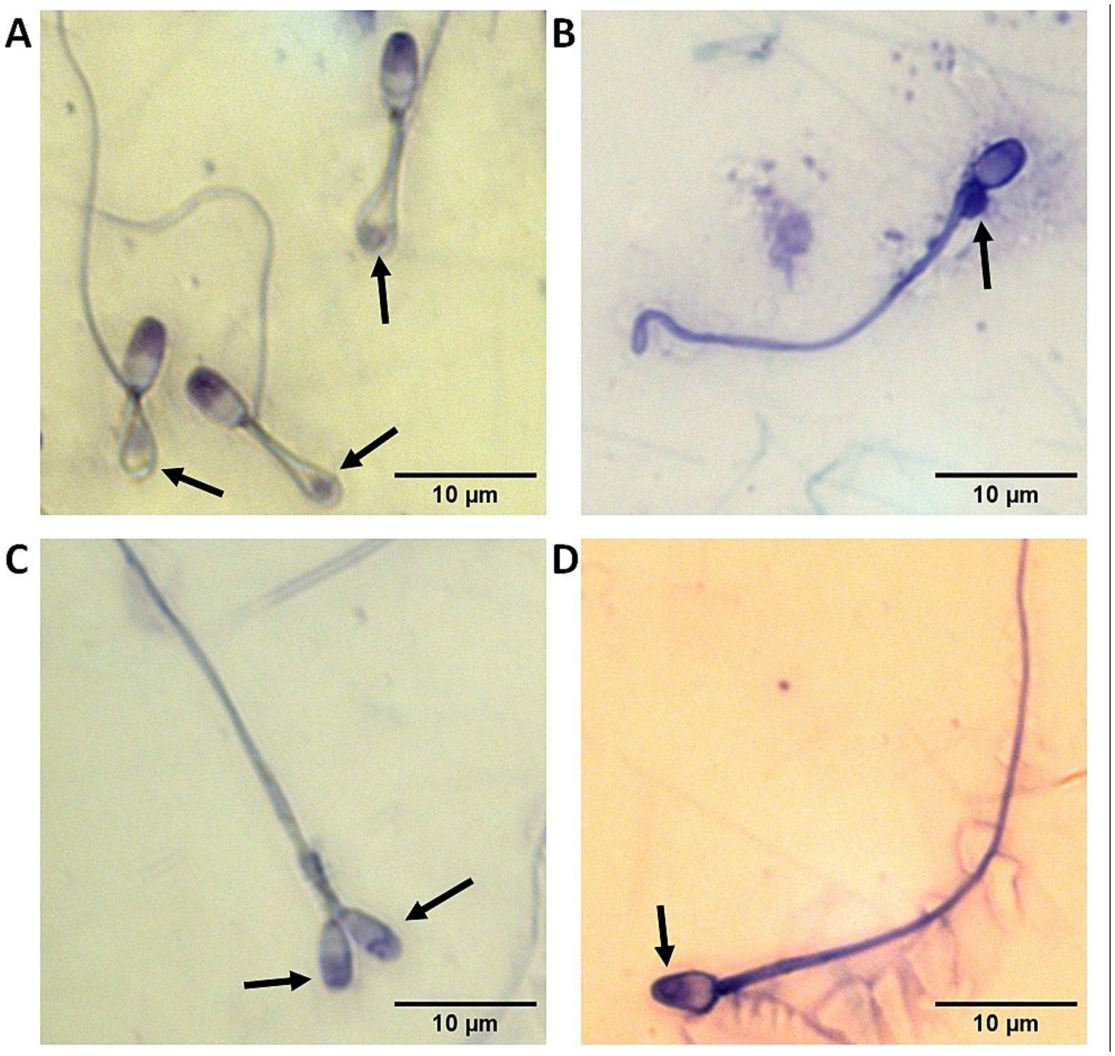
Representative images with black arrows pointing out at sperm abnormalities commonly observed in the domestic cat including **(A)** reflexed flagella with cytoplasmic droplets trapped inside the bends; **(B)** proximal cytoplasmic droplet; **(C)** two heads; and **(D)** abnormal acrosome.

Epididymides were removed from testes and rinsed in PBS. A small number of spermatozoa were collected from the cauda and assessed via Coomassie staining to assess sperm morphology and identify teratospermic individuals (≥60% abnormal spermatozoa; Figure 1). For each experimental replicate, cauda epididymides from 2 teratospermic cats (*n* = 22 total teratospermic cats) were placed in a petri dish on a warmer set to 38.5°C with supplemented Ham’s F-10 medium (Ham’s F-10 Nutrient Mix with HEPES supplemented with 1 mM pyruvate, 2 mM L-glutamine, 100 IU/mL penicillin, 100 μg/mL streptomycin, and 5% fetal bovine serum) and sliced with a scalpel blade. Sperm cells were allowed to seep out for 10 min before liquid was transferred to a 1.5-ml tube. Motility was visually assessed and recorded using a phase contrast microscope before centrifuging sperm cells at 300 *× g* for 8 min. The supernatant was removed, and the pellet was overlaid with 30 μL of fresh supplemented Ham’s F-10 medium (swim up procedure). The tube was placed in a 38.5°C incubator and left undisturbed for 30 min. The supernatant was then carefully collected, and motility was assessed and recorded again.

### 2.5 Incubation of sperm with extra-cellular vesicles prior to in vitro fertilization

After the swim up procedure, the concentration of the sperm sample was evaluated. Sperm were incubated in a rotating incubator at 38.5°C for 1 h and 15 min at a concentration of 30–50 million sperm/ml and supplemented with normospermic EVs at a final concentration of 4 μg/μl in supplemented Ham’s F-10 medium. We previously reported the use of this concentration and co-incubation duration. It was determined by assessing the optimal length of exposure which allowed for the uptake of EV proteins while simultaneously maintaining adequate sperm motility (21, 22). Control sperm from teratospermic cats were incubated under the same conditions but with an equal volume of Ham’s F-10 Nutrient Mix with HEPES without any normospermic EVs. At the end of the incubation, samples were centrifuged at 300 *× g* for 8 min. The supernatant containing the EVs that were not bound to the sperm was removed, and the pellet containing the sperm was resuspended in SAGE protein plus blastocyst medium. Sperm motility and concentration were assessed and recorded.

### 2.6 *In vitro* fertilization

Matured oocytes were washed free of IVM medium and placed in drops of SAGE protein plus blastocyst medium under mineral oil. Sperm cells were added to the IVF drops at a final concentration of 1 million motile sperm/ml. The dishes were allowed to incubate in an incubator at 38.5°C and 5% CO_2_ for up to 24 h before the oocytes were washed free of sperm. Cumulus cells were manually removed via The Stripper™ Pipettor (CooperSurgical), and oocytes were placed in fresh drops of SAGE protein plus blastocyst medium. The oocytes (presumptive zygotes) were cultured at 38.5°C in 5% CO_2_ for 7 days, replacing the culture medium on Day 3.

### 2.7 Staining and observations of oocytes and embryos

On Day 7 of culture, the oocytes/embryos were fixed with 4% paraformaldehyde for at least 30 min at room temperature, followed by washing and permeabilization with 0.5% Triton X-100 in PBS and mounting in hard-set VectaShield with DAPI (Vector Labs) to count blastomeres using a microscope fitted with epifluorescence.

### 2.8 Statistical analysis

Statistical analyses were performed in GraphPad Prism (version 6 for Windows). Normality was assessed using the Shapiro–Wilk test. Fisher’s exact tests were used to compare embryo outcomes between EV co-incubation groups and controls. ANOVA was used to compare percentages of sperm defects between sperm used for each replicate of IVF.

### 2.9 Experimental design

**Figure 2:**
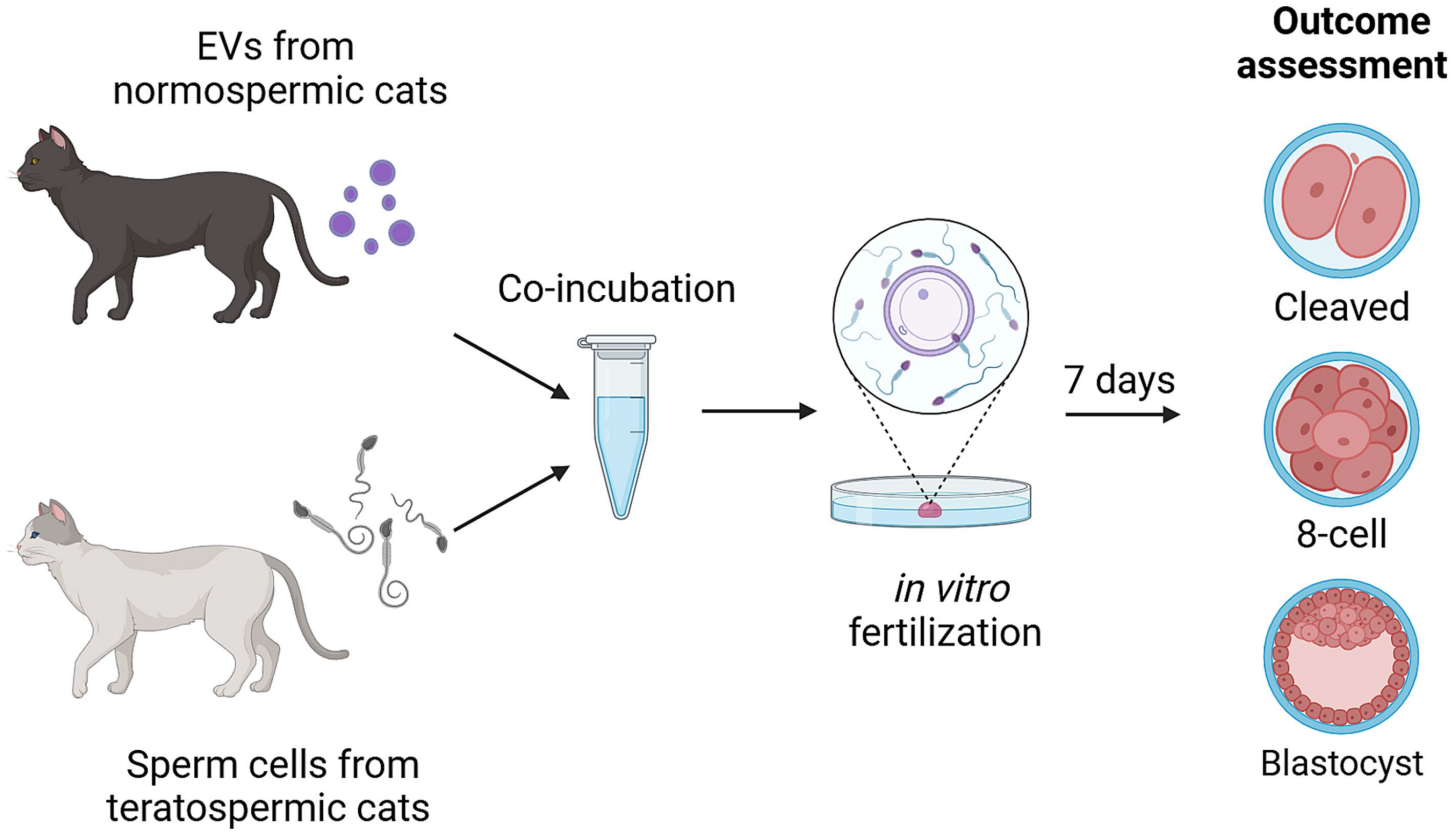
Experimental design showing incubation of sperm from teratospermic cats with EVs from normospermic cats prior to performing IVF and assessing for embryo cleavage, embryo development to at least the 8-cell stage, and development to blastocyst stage. Created in BioRender. Sosnicki, D. (2024) https://BioRender.com/b54x773.

Graphical overview of experimental design is summarized in Figure 2. Sperm from teratospermic cats (*n* = 2 males pooled together for each of the 11 experimental replicates) were incubated with EVs collected from normospermic cats (*n* = 5 cats pooled together for each of the 11 experimental replicates) for 1 h and 15 min prior to being added to matured oocytes (*n* = 233 total oocytes used for EV co-incubation across 11 replicates and *n* = 246 total oocytes used for controls without EV co-incubation across 11 replicates). Oocytes were fixed 7 days after fertilization and stained with DAPI to assess outcomes for percent of embryos that cleaved (experimental replicates 1–11), percent of embryos that progressed to at least the 8-cell stage of development (experimental replicates 5–11), and percent of embryos that progressed to the blastocyst stage of development (experimental replicates 5–11).

## 3 Results

### 3.1 Sperm morphology assessment

Sperm from the two teratospermic cats used for each experimental replicate were stained and assessed for morphologic abnormalities of the head and tail. Across all experimental replicates, the percentage of sperm with head defects ranged from 0 to 11%, and the percentage of sperm with tail defects ranged from 64.5 to 90% (see Supplementary Table 1 for results of individual experimental replicates).

### 3.2 Sperm motility assessment before/after swim up procedure and after co-incubation with or without EVs

Sperm motility after swim up for all replicates ranged from 85 to 95% (see Supplementary Table 2 for results of individual experimental replicates). After incubation without EVs the control sperm motility ranged from 55 to 85%, and after co-incubation with EVs the sperm motility ranged from 65 to 85% (see Supplementary Table 1 for results of individual experimental replicates).

### 3.3 Effect of EV co-incubation on embryo cleavage after IVF with teratospermic samples

**Figure 3:**
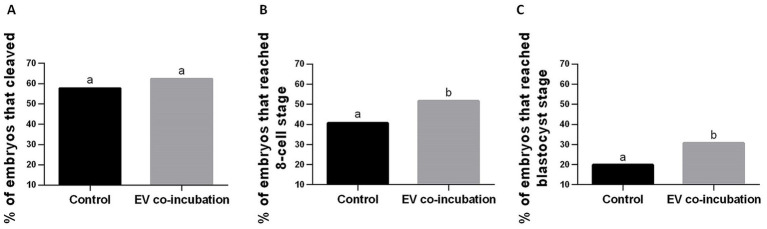
Percentage of embryos relative to the starting number of oocytes used for IVF in the control without EV co-incubation and in the EV co-incubation groups. **(A)** cleaved embryos in all replicates (replicates 1–11); **(B)** embryos that reached at least the 8-cell stage (replicates 5–11); and **(C)** embryos that reached the blastocyst stage (replicates 5–11). Different letters above bars indicate significant statistical differences between groups (*p* < 0.05).

Embryo cleavage was assessed as a proportion of all oocytes for experimental replicates 1–11 (see Supplementary Table 2 for results of each experimental replicate). There was a small but not significant (*p* = 0.15) increase in the percentage of embryos that cleaved in the EV co-incubation group (67.0%, 156 cleaved embryos out of 233 oocytes) compared to the control without EV co-incubation (60.6%, 149 cleaved embryos out of 246 oocytes; Figure 3A).

### 3.4 Effect of EV co-incubation on preimplantation embryo development after IVF with teratospermic samples

The percentage of embryos that reached at least the 8-cell stage was calculate as a percentage of all oocytes for experimental replicates 5–11 (see Supplementary Table 2 for results of each experimental replicate). The percentage of embryos reaching at least the 8-cell stage was significantly higher (*p* = 0.04) in the EV co-incubation group (58.4%, 97 embryos out of 166 oocytes) compared to the control without EV co-incubation (47.2%, 83 embryos out of 176 oocytes; Figure 3B).

### 3.5 Effect of EV co-incubation on blastocyst formation after IVF with teratospermic samples

The percentage of embryos that reached the blastocyst stage was calculated as a percentage of all oocytes for experimental replicates 5–11 (see Supplementary Table 2 for results of each experimental replicate). The percentage of embryos reaching the blastocyst stage was significantly higher (*p* = 0.02) in the EV co-incubation group (30.7%, 51 embryos out of 166 oocytes) compared to the control without EV co-incubation (19.9%, 35 embryos out of 176 oocytes; Figure 3C).

## 4 Discussion

Our findings indicate that, with these experimental numbers, there was not a significant effect of EV co-incubation on the percentage of embryos that cleaved. However, the percentage of embryos that reached at least the 8-cell stage was significantly higher after EV co-incubation, as was the percentage of embryos that developed into blastocysts after EV co-incubation. This indicates that changes induced by exposure to EVs from normospermic cats, and presumed transfer of contents to sperm cells, may more strongly affect the developmental potential of the sperm, rather than simply impacting its ability to fertilize or stimulate oocyte activation. In addition to our results that examined the effect of EV co-incubation on embryo outcomes of reaching at least the 8-cell stage and the blastocyst stage being statistically significant (*p* < 0.05), the difference of greater than 10-percentage points between the EV co-incubation groups (58.4% for 8-cell stage and 30.7% for blastocyst stage) and the control groups without EV co-incubation (47.2% for the 8-cell stage and 19.9% for the blastocyst stage) is also clinically significant.

It is interesting to note that there were variations in results between individual replicates regarding the number of embryos that reached the 8-cell and blastocyst stages (See Supplementary Table 2). For embryos that reached at least the 8-cell stage this ranged from a difference of only 8.5 percentage points between control and EV co-incubation groups in replicate 8, to a difference of 37.9 percentage points between control and EV co-incubation groups in replicate 11. For embryos that reached the blastocyst stage this ranged from a difference of 3.1 percentage points between control and EV co-incubation groups in replicate 5, to a difference of 35.3 percentage points between control and EV co-incubation groups in replicate 7. As different cats were used as the sources of sperm and EVs used for the experiments, this difference could mean that EV co-incubation is more effective at rescuing some specific types of sperm defects than others, or that differences existed among EVs from the normospermic cats.

**Figure 4:**
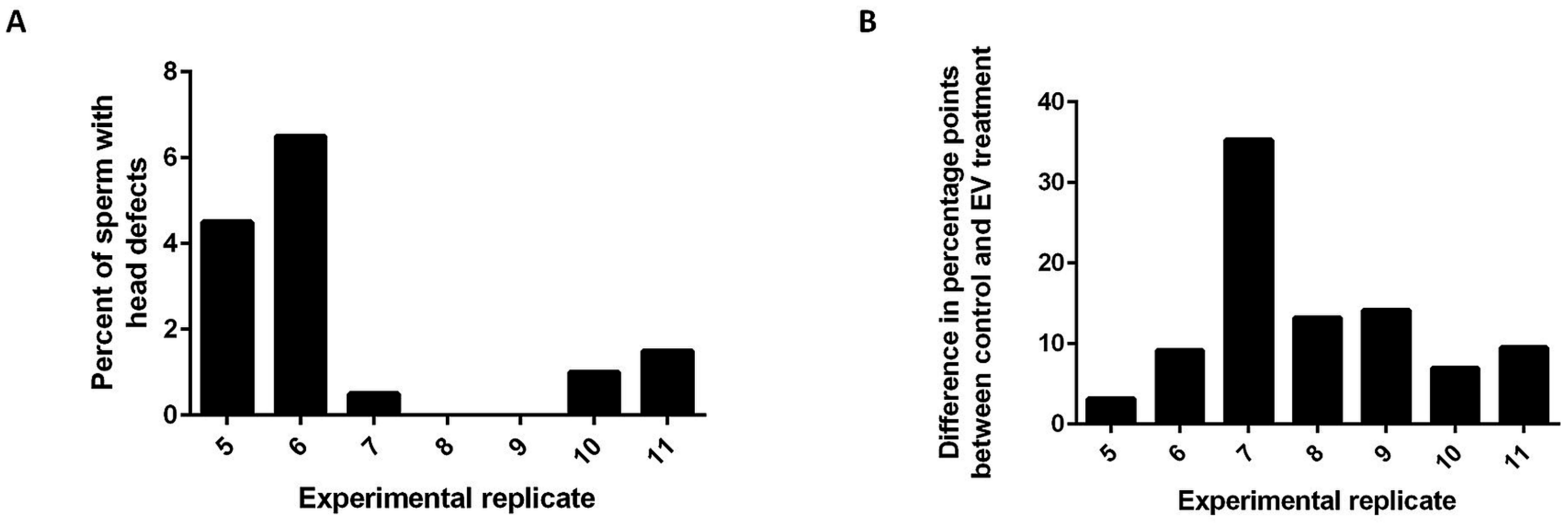
Histograms showing that EV co-incubation may be better at rescuing sperm function in samples containing fewer head defects. **(A)** percentage of sperm that had sperm head defects for experimental replicates 5–11 and **(B)** the difference in percentage points between the control and EV co-incubation group for experimental replicates 5–11.

In the domestic cat, defects in spermatozoa are often attributed to several types of dysfunctions. Head, acrosome, and midpiece defects are thought to stem from testis dysfunction or dysregulation, and tail defects result from epididymal dysfunction (23). In this study, we pooled fresh sperm from 2 teratospermic males for each experimental replicate in an attempt to help minimize variation between individual cats, but it is possible that sperm from one individual contributed more than the other to the pool and that the type of morphological abnormalities were different between the two individuals. Similarly, the EVs were pooled from 5 normospermic cats for each experimental replicate to reduce the effect of individual differences, but the degree of variation in the composition of EV content among normospermic individuals is not known. Additional analyses of the types of morphological defects in the sperm from teratospermic cats that were used for each of the experimental replicates revealed that on average there were more tail defects (79% of sperm) than head defects (4.2% of sperm). While there was no statistical difference in the average number of head or tail defects seen between the experimental replicates (*p* > 0.05, ANOVA), experimental replicates that had the lowest prevalences of head defects [replicates 7 (0.5%), 8 (0%), and 9 (0%)] tended to have the greatest improvement in blastocyst outcomes when comparing the EV co-incubation to the controls without EV co-incubation [replicates 7 (35.3%), 8 (13.2%), and 9 (14.1%); Figure 4]. This trend may indicate that the EVs are better to “rescue” sperm with fewer head defects, indicating dysfunction of the epididymis and not the testis. However, more experiments are required to explore if incubation with EVs from normospermic cats may help to recapitulate a portion of epididymal maturation.

We chose the 8-cell stage of development as an assessment point for developmental potential because it has been reported that embryonic genome activation (EGA) occurs at or before this point in embryo development in the domestic cat (24, 25). Therefore, reaching at least the 8-cell stage of development is indicative that products of transcription of the embryonic genome, with contributions from the sperm, are required for continued development and maternal genome products are no longer sufficient to support development past this stage. Any differences observed between our control without EV co-incubation and EV co-incubation groups are indicative of improved developmental potential due to sperm-related factors, and in this study, those are attributed to components originating in the EVs derived from normospermic cats. We also attempted to compare gene expression of several developmentally important genes [DNA methyltransferases 1 and 3A, *DNMT1* and *DNMT3A*; gap junction protein *α* 1, *GJA1*; transcription factor octomer 4, *POU5F1* (*OCT4*); and insulin-like growth factor 1 and 2 receptors, *IGF1R* and *IGF2R*] that were previously described to establish the dynamic and temporal patterns of EGA in the domestic cat (25) using quantitative, real-time PCR (RT-PCR) to determine if there were also changes in the expression of any of the genes specifically due to EV co-incubation. Unfortunately, due to our sample sizes and the low amounts of RNA that are present in early embryos, we did not have enough biomaterial to optimally perform these experiments. However, in our preliminary RT-PCR experiments, we did not observe any differences in expression of these genes based on EV co-incubation. Additional experiments with increased sample sizes and increased RNA yields are needed to adequately determine if there is an effect of EV co-incubation on early embryo gene expression.

While we have previously reported a difference in the proteomic profiles of EVs derived from normospermic cats compared to those derived from teratospermic cats (17), the exact component(s) of the EVs that are responsible for the difference we are seeing here has yet to be identified. Epididymal EVs are known to transfer different types of components to sperm during epididymal transit, so although a protein or combination of proteins may be responsible for the difference we are seeing, it is also possible that the improvement is due to other combination of proteins, lipids, and/or microRNAs. For example, in epididymal segment 2 of the mouse, we have observed that the ganglioside G_M1_ is transferred to sperm through epididymosomes (11). Nonetheless, our proteomic profiling suggests that there could be a difference in the general composition of the EVs that are produced in normospermic cats versus those produced in teratospermic cats. Given the critical importance of epididymal sperm maturation and its dependance on the epididymal milieu, our findings suggest that the compositions of epididymal EVs may be partially responsible for the difference in quality of spermatozoa between normospermic and teratospermic individuals.

Further studies are warranted to pinpoint specific factors that affect specific changes in the early embryo that occur as a result of EV co-incubation, and also to identify changes directly to the sperm as a result of EV co-incubation. Importantly, findings of this study may help to improve assisted reproductive technologies used for wildlife conservation as the other species in the Felidae family also exhibit high incidence of teratospermia. Species with similar sperm traits to the domestic cat may benefit from the use of EVs from normospermic individuals as a treatment prior to assisted reproduction procedures (26). Compatibility between related species suggests the possibility of using biological material from common or domestic species to improve the sperm of wild and endangered species prior to using it for assisted reproduction procedures. For example, it could be possible to use EVs from normospermic domestic cats to improve the function of sperm from cheetah, clouded leopard, or tiger, which improves the practicality of such procedures since the domestic cat is widely abundant and epididymides needed for the collection of EVs could easily be collected during routine neuter procedures.

## Data Availability

The original contributions presented in the study are included in the article/Supplementary material, further inquiries can be directed to the corresponding author.

## Footnotes

^1^ https://evtrack.org/
